# Mortality Pattern of *Poecilus cupreus* Beetles after Repeated Topical Exposure to Insecticide—Stochastic Death or Individual Tolerance?

**DOI:** 10.1021/acs.est.3c08031

**Published:** 2024-01-22

**Authors:** Grzegorz Sowa, Agnieszka J. Bednarska, Ryszard Laskowski

**Affiliations:** † Institute of Environmental Sciences, Jagiellonian University, Gronostajowa 7, 30-387 Kraków, Poland; ‡ Institute of Nature Conservation, Polish Academy of Sciences, A. Mickiewicza 33, 31-120 Kraków, Poland

**Keywords:** Carabidae, agriculture, insecticides, habitats, mortality pattern

## Abstract

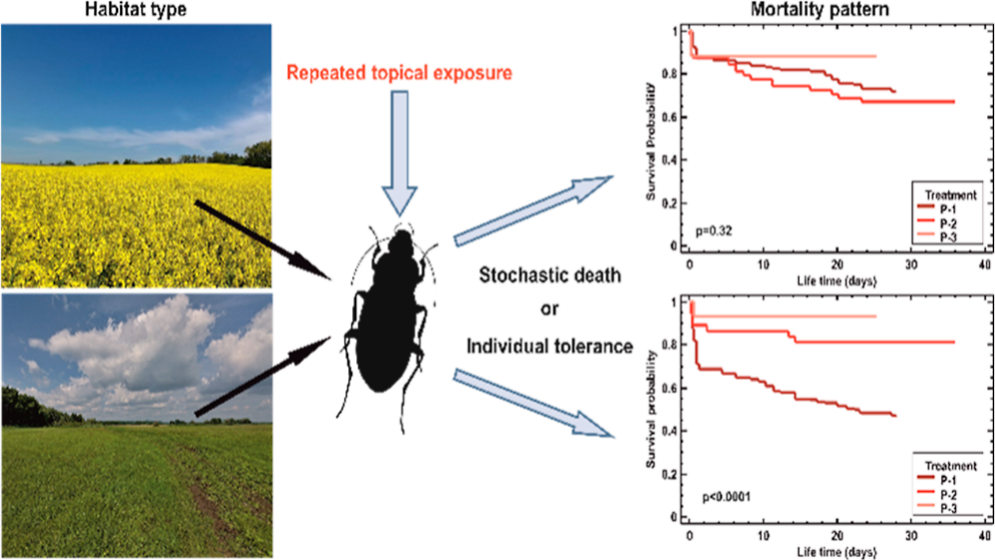

The mortality of organisms exposed to toxicants has been attributed to either stochastic processes or individual tolerance (IT), leading to the stochastic death (SD) and IT models. While the IT model follows the principles of natural selection, the relevance of the SD model has been debated. To clarify why the idea of stochastic mortality has found its way into ecotoxicology, we investigated the mortality of *Poecilus cupreus* (Linnaeus, 1758) beetles from pesticide-treated oilseed rape (OSR) fields and unsprayed meadows, subjected to repeated insecticide treatments. We analyzed the mortality with the Kaplan–Meier estimator and general unified threshold model for survival (GUTS), which integrates SD and IT assumptions. The beetles were exposed three times, ca. monthly, to the same dose of Proteus 110 OD insecticide containing thiacloprid and deltamethrin, commonly used in the OSR fields. Kaplan–Meier analysis showed that the mortality of beetles from meadows was much higher after the first treatment than after the next two, indicating the IT model. Beetles from the OSR displayed approximately constant mortality after the first and second treatments, consistent with the SD model. GUTS analysis did not conclusively identify the better model, with the IT being marginally better for beetles from meadows and the SD better for beetles from OSR fields.

## Abstract 2

Although the mortality pattern may resemble a stochastic process in populations with reduced genetic variance in sensitivity, individual sensitivity governs the mortality of organisms exposed to insecticides.

## 1 Introduction

Toxicokinetic–toxicodynamic (TK–TD) models play a crucial role in predicting the effects of toxic chemicals on organisms under different exposure scenarios, including fluctuating or pulse exposures.^1^ These models are highly valuable in ecotoxicological research and environmental risk assessment of chemicals.^2−4^ However, developing effective TK–TD models requires a comprehensive understanding of the processes that lead to mortality of individuals when exposed to toxic chemicals. This has been a subject of extensive debate in ecotoxicology, with two contrasting hypotheses, namely, the individual tolerance (IT) and stochastic death (SD) models, attempting to explain this phenomenon.^5,6^

The IT model, also referred to as the individual effect dose (IED), forms the fundamental assumption of well-established methods used to analyze the dose/concentration–response relationship, such as probit and logit analysis. The IT approach takes into consideration the inherent differences in the sensitivity of individuals to chemical stress. It acknowledges that each individual has a specific threshold of tolerance, and when the damage caused by stress surpasses its threshold, the individual dies. In this approach, death is seen as an individual-specific response rather than a gradual probabilistic process. The SD approach assumes that every individual within a population shares the same threshold, in terms of susceptibility to the chemical stressor and faces a certain probability of dying as a result of exposure to that stressor. This probability of death increases progressively as the extent of damage caused by the stressor increases and surpasses a specific threshold. In essence, the SD approach views death as a probabilistic event, becoming more likely as the level of stress-induced damage exceeds a critical point. The SD model challenges the notion of individual variation in tolerance and argues for a more probabilistic understanding of mortality in response to toxic exposure.^7^

The ongoing debate between scientists about the adequacy of the IT and SD models reflects the complexity of ecotoxicological phenomena and the challenges in accurately predicting the effects of toxicants on organisms. For the last two decades, researchers continued to explore these models to improve our understanding of the underlying mechanisms.^8^ From a practical point of view, both models appeared useful in assessing the environmental risks posed by chemicals,^9^ but they represent two contrasting hypotheses that aim to explain dose–effect relationships in ecotoxicology. Although the IT model has been used for decades, it has been challenged by several studies (e.g., refs and (7) and (10)–^11^^12^^13^). However, evidence that mortality itself is not a stochastic process, but rather is determined by the genetically determined distribution of susceptibility in a population, is well demonstrated by the evolution of insecticide resistance in many pest populations.^14,15^ The IT model is also consistent with the central assumption of modern biology that the values of essentially all traits in a population, including resistance to environmental factors, have approximately normal or log–normal distributions. If this were not the case, evolution based on “survival of the fittest” would not be possible.^16,17^

So why have some researchers proposed a hypothesis that contradicts the foundations of modern biology? Following the arguments put forward by the researchers,^7,10^ it can be assumed that the SD model was proposed as a result of flawed research or errors in interpreting the results. In fact, it is not difficult to come up with studies suggesting that mortality from exposure to a toxic substance is a random process—it is enough that the experimental population is largely devoid of natural genetic variation. In such populations with negligible variance in the sensitivity to toxicants, the mortality pattern may indeed resemble a stochastic phenomenon. Since ecotoxicological bioassays are usually performed on laboratory-grown cultures of experimental organisms (sometimes even clones), this explanation seems very plausible.^18^ Similar results might also be obtained for field populations preselected for increased resistance through repeated insecticide applications. However, even in such cases, we encourage scientists to consider genetically determined variation in fitness, which is consistent with the paradigm of biology. Moreover, the parameters estimated on laboratory populations are intended to help us predict the effects of exposure to toxic substances in wild populations, which tend to have greater genetic variability than laboratory cultures.

The IT and SD models have been integrated into the general unified threshold model for survival (GUTS), which has been proposed as a comprehensive framework focused on survival bioassays in ecotoxicological risk assessment (ERA).^6^ Initially proposed by Jager et al.^6^ and refined by Jager and Ashauer,^19^ GUTS has gained recognition as a valuable tool for evaluating the potential impacts of various stressors, with a particular emphasis on pesticides.^20^ The endorsement of GUTS by the European Food Safety Authority^5^ and OECD^21^ marked a significant milestone for TK–TD modeling and suggests that both SD and IT models should always be used unless one of them clearly does not describe data well. Furthermore, EFSA recognized the importance of GUTS in pesticide risk assessment, stating that it is “ready to be used” in regulatory practices.^5^ This recognition has provided a substantial boost to the application of GUTS in routine assessments performed by regulatory agencies. The GUTS encompasses several different models, namely, GUTS-SD, GUTS-IT, GUTS-RED-IT, and GUTS-RED-SD, which are designed to provide different approaches to understanding the relationship between exposure, damage, and survival.^22^ While we admit that GUTS can be a useful tool for ERA, care has to be taken if the estimated parameters are to be used for predicting population responses in their natural environment. Furthermore, we insist that conclusions should not be drawn from the GUTS-SD model about the nature of the death processes.

In conventional agriculture, virtually all crops are treated with pesticides, often several times during the growing season.^23^ Consequently, in agricultural areas, not only pests but also nontarget arthropods (NTAs), including those providing important ecosystem services (e.g., pest control or pollination), are exposed to pulsed pesticide exposures. Hence, ecological risk assessment for pesticides should assess not only the effects of a single exposure but also repeated exposures. The accumulation of pesticides and pesticide-driven damage and the repair of that damage can be fast or slow, depending on the substance properties, mode of action, and species. If pesticides and damage accumulate faster than an organism can recover from each treatment, then the toxic effects of repeated treatments can be magnified due to the possibility of transferring the toxic effects of the previous exposure to the next exposure. Consequently, each subsequent exposure can lead to an even higher mortality rate than the previous one.^24,25^ On the other hand, repeated exposures to pesticides may result in the selection of the most resistant individuals, thus reducing the overall variance in individual susceptibility to pesticides. If a population exposed to such repeated exposures is then tested for the effects of pesticides, this could lead to the false conclusion that mortality is a stochastic process, because—due to the low variance in sensitivity—an approximately constant proportion of the population would be killed by subsequent pesticide sprays. In contrast, in the IT model, the mortality rate (fraction killed) should decrease with subsequent exposures because the least tolerant individuals would be eliminated from the population with the first spray. This reasoning offers an excellent experimental design for testing the SD vs IT hypothesis.

In the present study, we aimed to evaluate the two alternative mortality models—the IT and SD—by comparing the mortality of carabid beetles, *Poecilus cupreus* (Linnaeus, 1758), from pesticide-treated oilseed rape (OSR) fields or untreated meadows after repeated exposure to an insecticide Proteus 110 OD [active ingredients thiacloprid (100 g L^–1^) and deltamethrin (10 g L^–1^)] in the laboratory. Carabids, which are important NTAs and ecosystem service providers (ESPs), are sensitive to a wide range of insecticides,^26,27^ with adverse effects observed on their abundance and diversity^28^ as well as physiological and biochemical processes.^29^ The main goal of this study was to determine whether the observed mortality patterns better fit the IT or SD models by analyzing mortality data using standard Kaplan–Meier survival analysis and by fitting IT and SD models as implemented in GUTS-RED.^22^ We expected that populations from meadows, presumably not being exposed previously to insecticides, would show a higher variance in mortality after insecticide exposure in the laboratory due to the lack of selection for resistance, while populations from OSR fields, most likely being exposed previously to insecticides, would show increased mean tolerance and a lower variance in mortality after insecticide exposure in the laboratory. We hypothesized that, with this setup, we should be able to show that while in populations from meadows, mortality follows the IT model (i.e., decreasing mortality rate with successive treatments), the populations from OSR may exhibit a mortality pattern resembling the SD model.

## 2 Materials and Methods

### 2.1 Study Area and Site Selection

**Figure 1:**
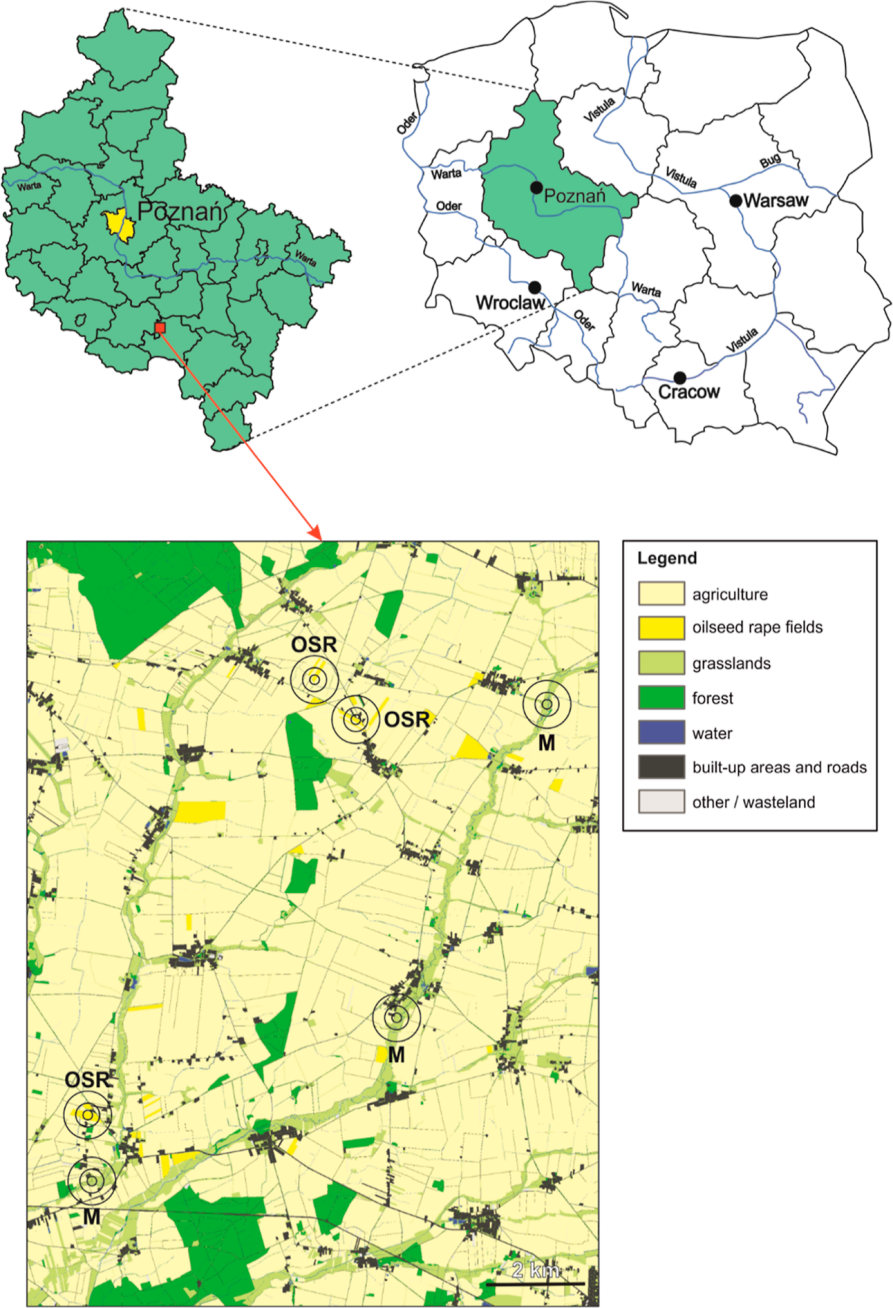
Location of the trapping area. Upper panel: on the left—the administrative division of Wielkopolska Province with its capital (Poznań) marked in yellow and the location of the study area marked with a red square; on the right—an administrative division of Poland into provinces with Wielkopolska Province marked with green. Lower panel: locations of the study sites (M—meadow, OSR—oilseed rape) marked by circles with a different buffer zone—100, 250, and 500 m radius.

The beetles were collected from a farmland area in the southwest part of the Wielkopolska province in Poland (Figure 1, Supporting Information 1) during their peak activity period, which occurs between April and May, and within a minimum period of 2 weeks between spraying by farmers. Two different habitat types were sampled: meadows (M), representing seminatural habitats untreated with pesticides, and OSR fields, where pesticides were regularly used. To ensure an adequate number of beetles and to represent the habitat type rather than just a single site or population, the beetles were collected from three meadows and three OSR fields (Figure 1), and all beetles from one habitat type were combined for the experiment. Sixty-four Barber traps without any preservative, arranged in an 8 × 8 m grid and covering an area of 64 m^2^, were set up in the middle of each site. In OSR fields, the OSR coverage was approximately 35 to 91% within a 100 m radius, 16 to 49% within a 250 m radius, and 10 to 15% within a 500 m radius around the midpoint where the beetle traps were located (Figure 1). On the other hand, the traps in the meadow sites were located in natural grasslands, not intensively cultivated, except for occasional mowing. Furthermore, there were no OSR fields within a 500 m radius of the traps (Figure 1). Previous studies indicated that the buffer zone of a 500 m radius is sufficient to prevent significant migrations between local populations.^30,31^ The traps were emptied every 2–3 days, and the collected beetles were sorted directly in the field. Collected *P. cupreus* beetles were placed in plastic containers (23 × 17 × 11 cm) filled with moist peat and transported to the laboratory, where they were kept at 20 ± 2 °C, 70 ± 5% relative humidity, and the light–dark regime 16 h light:8 h darkness.

### 2.2 Study Species

The ground beetle *P. cupreus* was selected for this study due to its abundance across all study sites. This species is widely distributed and considered one of the most common and dominant carabid beetles found in agricultural areas throughout Europe.^26^ Furthermore, it serves as a prime example of a beneficial predator that plays a crucial role in the ecosystem by providing pest control services.^32^ Adult *P. cupreus* beetles are primarily active during the day and disperse mostly by walking, although they can fly occasionally.^33^ While they generally do not exhibit extensive movement throughout their active period, which occurs during spring and summer, observations have revealed that on average, they can displace ca. 3–30 m daily, and a monthly displacement is in the range of 45–250 m.^26^ The extent of their dispersal depends on factors such as the composition of the landscape and the availability of resources.^34^

The species is associated with various crops, with a particular affinity for OSR as a favorable habitat during the spring.^35^ Additionally, it can be found in different types of meadows characterized by relatively high soil moisture.^36^ It is a “spring breeder”, with the main reproductive period occurring from April to the end of July. Following the reproductive phase, new generation adults emerge in August, and by late September, the beetles enter diapause to overwinter.^37^ Due to their lifespan, adult beetles can experience two distinct activity periods: the first shortly after hatching, just before entering winter diapause, and the second, longer period in the following spring and summer. This reproductive strategy allows them to maximize their population size and contribute to their ecological role as effective predators and pest control agents.

### 2.3 Experimental Design

**Figure 2:**
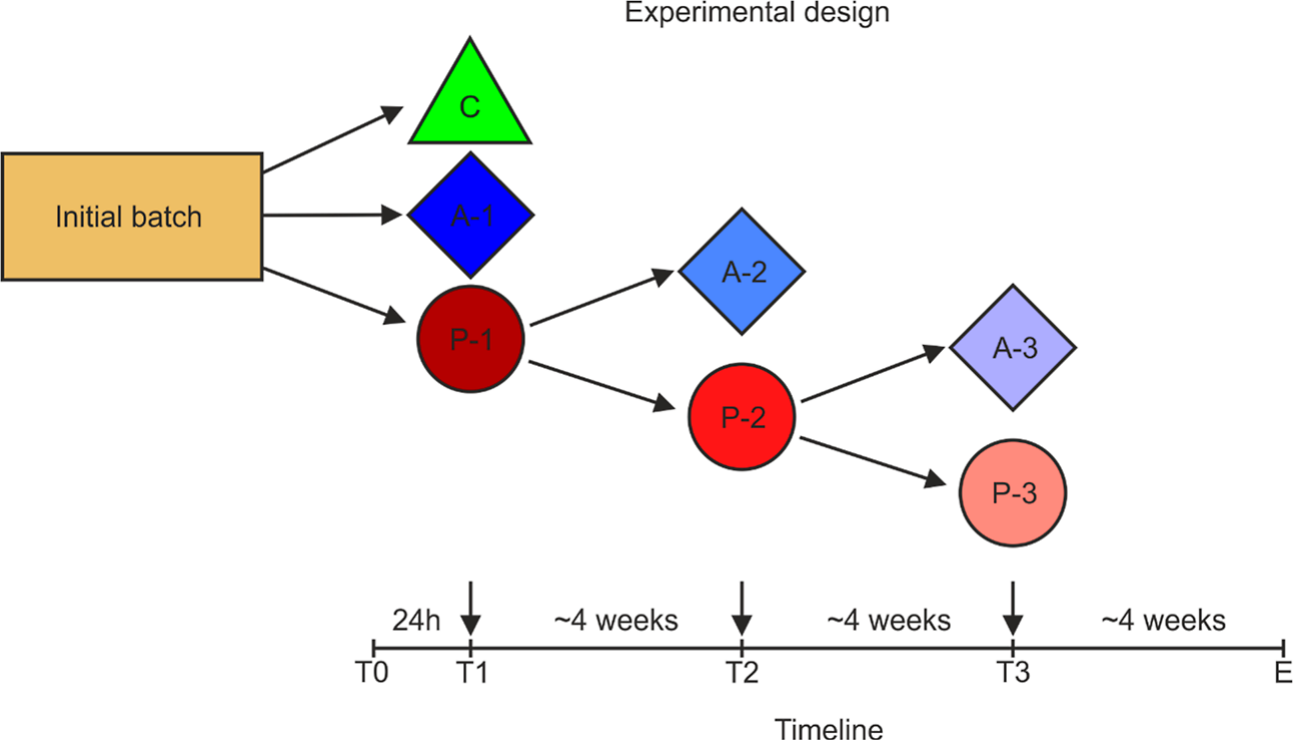
Diagram showing the experimental sequence of each group. C—control beetles without any exposure, A-1, A-2, A-3—solvent control groups with a single acetone application, P-1, P-2, and P-3—beetles treated with the insecticide Proteus 110 OD. The timeline represents the course of the experiment; T0—day zero when the beetles were placed in the Petri dishes, T1, T2, and T3—consecutive treatments, and E—the end of the experiment. The black arrows on the timeline indicate topical dosing of the insecticide.

Overall, 480 individuals, both males and females, were used in the experiment. Initially, the beetles were divided into three treatment groups within each habitat type: a control group without any application (40 beetles per habitat, control), a solvent control group with a single acetone application (40 beetles per habitat, acetone or A-1 group), and a group treated with the insecticide Proteus 110 OD (160 beetles per habitat, P-1). The beetles in the insecticide group were treated three times, with each treatment administered approximately every 4 weeks. Before each consecutive treatment, the surviving beetles from the previous insecticide treatment were randomly divided into two new groups, each assigned to either another round of insecticide treatment or acetone application (P-2 or A-2, and P-3 or A-3, respectively, after the second and third treatments, Figure 2).

Proteus 110 OD was chosen due to its frequent usage in the area (as indicated by a survey on pesticide usage conducted among local farmers). It consists of two active ingredients: thiacloprid (100 g L^–1^) and deltamethrin (10 g L^–1^). Thiacloprid belongs to the family of neonicotinoids, which affect the insect nervous system by stimulating nicotinic acetylcholine receptors. Deltamethrin is a pyrethroid that prevents the closure of voltage-gated sodium channels in axonal membranes, leading to dysfunction of spiracles and eventual insect death caused by desiccation. The commercial formulation of Proteus 110 OD was dissolved in acetone to achieve a concentration equivalent to 30% of the recommended field application concentration for OSR pests (application of 0.6 L of the product in 300 L of water per hectare is recommended). The concentration was chosen based on our previous experiments to be strong enough to give a clearly visible effect in terms of the percentage of individuals killed or knocked down while allowing enough beetles to survive until the next dose.

Twenty-four hours before application of the insecticide or acetone (T0), the beetles were individually placed in 35 mm-diameter plastic Petri dishes (FL Medical, Italy) for acclimatization. Subsequently, they were exposed individually to the insecticide or acetone using the standard topical exposure method^38,39^ by applying a 1 μL droplet of the solution to the scutellum using a Hamilton syringe with a repeater (Hamilton Company, USA). The doses of thiacloprid and deltamethrin per beetle were 0.06 and 0.006 μg, respectively. All insecticide-treated beetles therefore received an identical amount of insecticide solution, and to ensure that the entire dose penetrated the body, the beetles were immobilized until the acetone had completely evaporated. The beetles were fed *ad libitum* every third day with an artificial diet made of ground mealworm (*Tenebrio molitor*) larvae mixed with ground apples, according to the method described by Bednarska and Laskowski,^40^ but without any added preservatives to eliminate contact with potentially harmful chemicals. Mortality and immobility of the beetles were recorded after 2, 4, 6, 8, 10, 12, and 24 h, followed by daily observations for the entire period after each treatment. The experiment ended 4 weeks after the third treatment (see Supporting Information 2).

The beetles were not weighed, but our previous study on populations originating from the very same habitats showed no differences in body mass between beetles from meadows and OSR fields.^41^ Moreover, before starting the experiment, those beetles from each site which were not used in the experiment were killed by freezing, dried at 110 °C, and weighed to the nearest 0.0001 g (Radwag XA 110/2X, Poland). One-way ANOVA confirmed that the dry body mass of beetles did not differ significantly between sites (*p* = 0.38). As adult beetles do not grow, and changes in their body mass, if any, can be observed mostly just after overwintering,^42^ which was not the case in our study, we assumed that with food provided *ad libitum* their body mass remained constant over the experiment.

### 2.4 Statistical Analysis

#### 2.4.1 Kaplan–Meier Analysis

The survival probability of beetles was assessed separately for each stage of the experiment (i.e., after each treatment) and for each habitat type (meadow or OSR) using Kaplan–Meier survival analysis. Beetles that survived until the next treatment or the end of the experiment were censored. The potential impact of the solvent was examined by comparing the survival curves of untreated beetles (control) with those treated with acetone (acetone, A-1) during the initial 4 weeks of the study.

The survival rates following each consecutive exposure were first compared within each of the two groups: the acetone-treated beetles (A-1 vs A-2 vs A-3) and the pesticide-treated ones (P-1 vs P-2 vs P-3) for each habitat type separately. Then, survival curves after each consecutive dose of Proteus 110 OD were compared to their respective acetone treatments (i.e., P-1 vs A-1, P-2 vs A-2, P-3 vs A-3) for each habitat type separately. Additionally, comparisons of survival curves between habitat types within individual sprays were performed (i.e., OSR A-1 versus meadow A-1, and OSR P-1 versus meadow P-1). The statistical comparison of Kaplan–Meier survival curves was performed using the Wilcoxon test, with a significance level set at *p* ≤ 0.05. The selection of the Wilcoxon test was based on its suitability for situations where the hazard ratio is higher during earlier survival times compared to later ones,^43^ which is typically observed following insecticide exposure.^44^

This part of the statistical analysis was conducted using a Statgraphics Centurion 19 (Statgraphics Technologies, Inc., USA).

#### 2.4.2 GUTS Analysis

The same data set was analyzed with openGUTS (ver. 1.1). Following EFSA^3^ recommendation, we decided to use both the IT and SD models (GUTS-RED) implemented in openGUTS to test the extent to which each model can describe survival pattern in each population after the consecutive pesticide doses. To meet openGUTS requirements, the data first had to be restructured: instead of the lifetime of individuals as used in Kaplan–Meier analysis, the number of alive individuals was reported each day after the treatment, starting at 0.5 day, and the number of survivors after the second (P-2) and third (P-3) treatments was recalculated to account for the 50% of individuals that were used as the respective solvent control groups (A-2 and A-3) after subsequent doses. The background hazard was prefitted to the acetone control for each habitat type (meadow or OSR), where the survival data were also combined from the three consecutive solvent control groups after each treatment. In that way, the mortality after each insecticide dose was corrected for the respective background mortality of the beetles from the same habitat type.

As the beetles were exposed to the insecticide using topical application, we assumed a single-point exposure, meaning that the whole dose penetrated the beetle body in a short time. Specifically, in the GUTS model, a dose of 30% of the recommended concentration for field application was assigned to days 0, 29 and 66, when it was applied, with a dose of zero on all other days of the experiment, starting on days 0.5, 29.5, and 66.5. The assumption of fast penetrations of the insecticide into the beetle body was confirmed by the very high mortality in the first 24 h after the treatment, as seen especially in the beetles from meadows after the first dose.

## 3 Results

### 3.1 Kaplan–Meier Analysis

**Figure 3:**
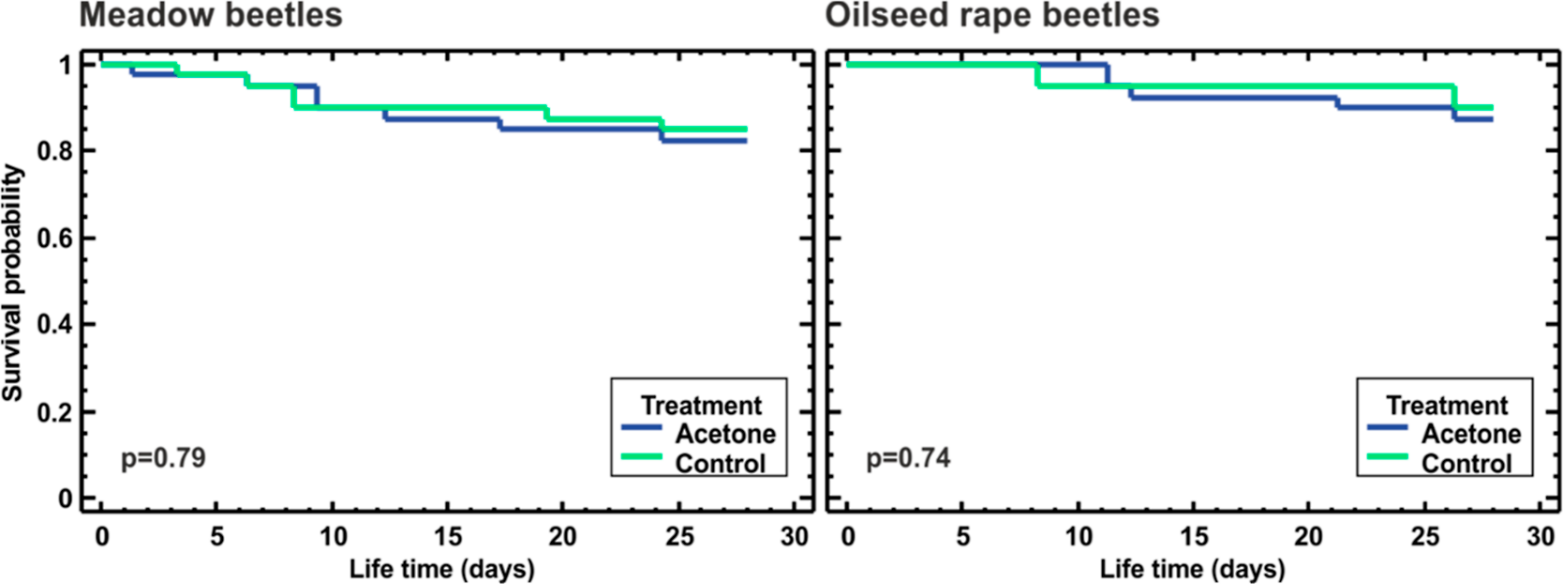
Survival of *P. cupreus* originating from meadows (left) and OSR fields (right), observed for 4 weeks after topical treatment with 1 μL of acetone (dark blue line, acetone) or not treated (green line, control); no significant differences between the treatments were found (*p* values indicated on the plots, Wilcoxon test).

**Figure 4:**
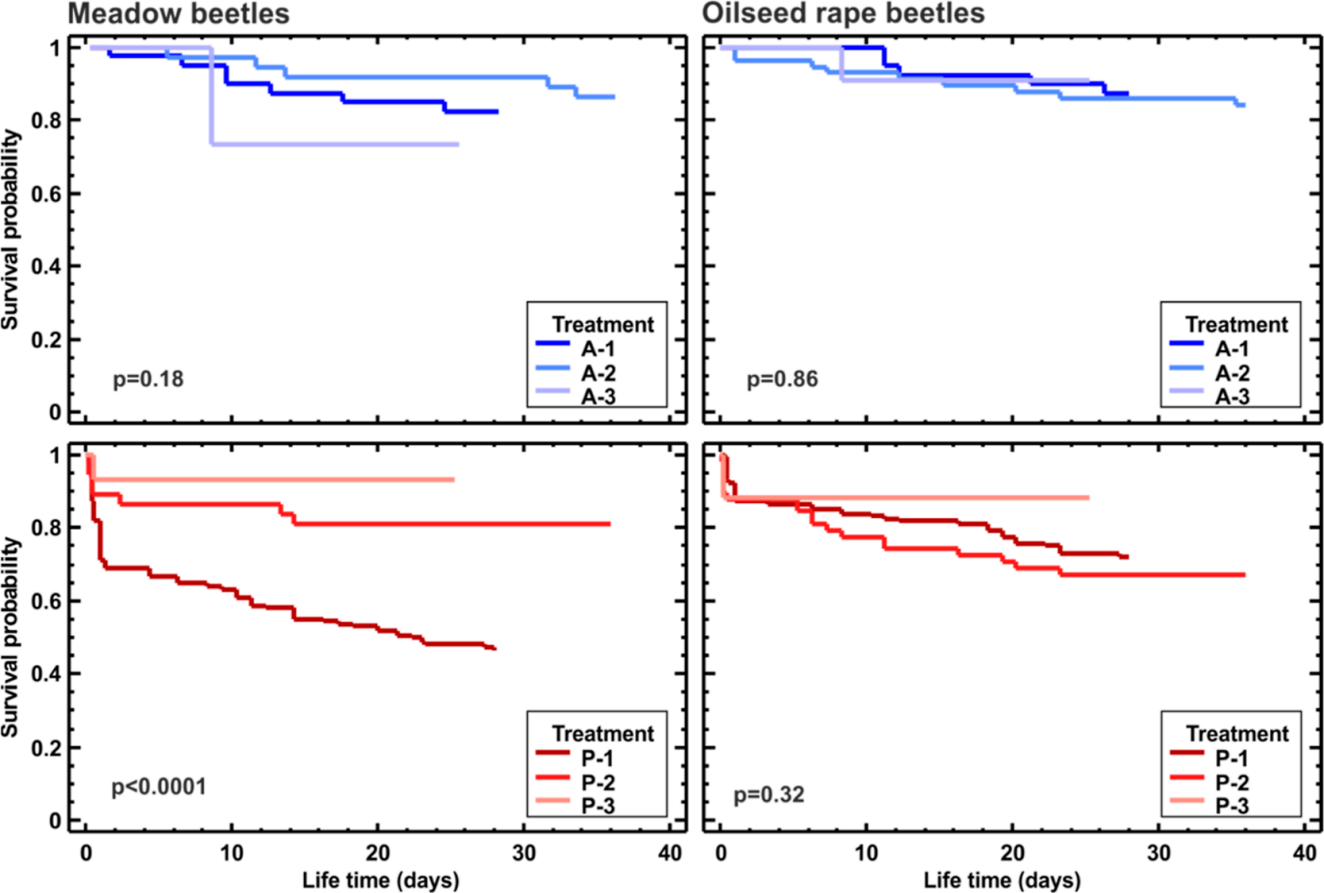
Survival curves of *P. cupreus* originating from meadows (left column) and OSR fields (right column) after exposure to three consecutive doses of Proteus 110 OD (P-1, P-2, and P-3; bottom row) and their respective acetone treatment (A-1, A-2, and A-3; upper row). Differences between survival curves tested with Wilcoxon test; *p* values indicated on the plots.

The survival curves of beetles from both control treatments (i.e., not treated and treated topically with 1 μL of acetone) for the two analyzed habitats did not differ from each other (*p* = 0.79 and *p* = 0.74 for beetles from meadows and the OSR fields, respectively, Figure 3). The comparison of the three acetone treatments showed no difference in survival for either beetles from meadows or those from the OSR fields (*p* = 0.18 and *p* = 0.86, respectively; Figure 4, top row).

The comparison of survival curves between consecutive insecticide treatments revealed no difference in survival for beetles from OSR fields (*p* = 0.32) and significant differences for beetles from meadows (*p* < 0.0001) for which the mortality was the highest after the first treatment (P-1), lower after the second (P-2), and the lowest after the third (P-3) (Figure 4, bottom row).

**Figure 5:**
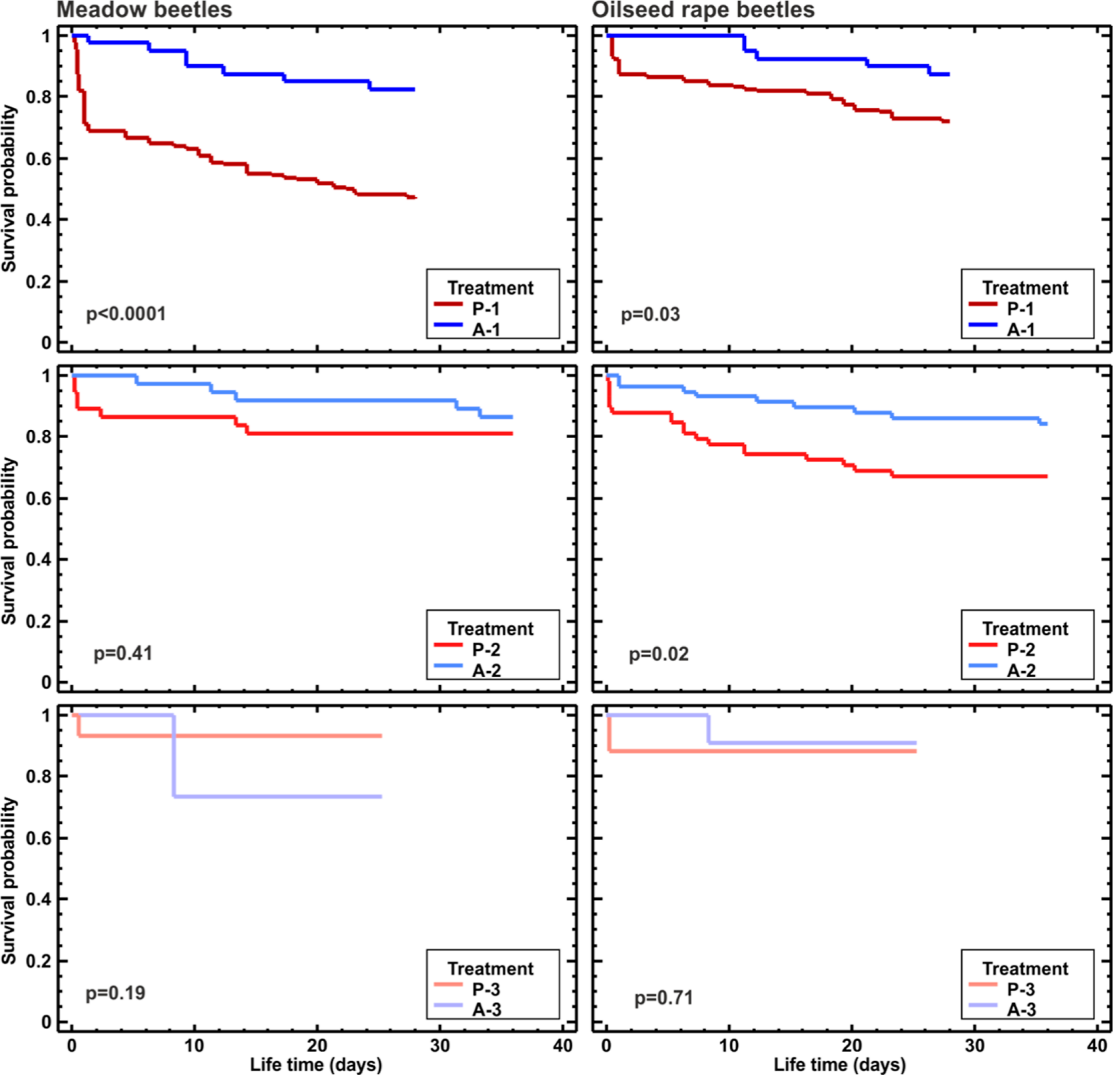
Survival curves of *P. cupreus* originating from meadows (left column) and OSR fields (right column) after three consecutive doses (P-1, P-2, P-3) of Proteus 110 OD (dark orange line) compared against respective acetone treatments (cyan line). Differences between survival curves tested with Wilcoxon test; *p* values indicated on the plots.

In pairwise comparisons of acetone vs insecticide treatments for beetles from meadows, a significant negative effect of insecticide was found for the first treatment (*p* < 0.0001) but not for the second and third treatments (*p* = 0.41 and *p* = 0.19, respectively; Figure 5, left column). For beetles from the OSR fields, both the first and the second insecticide treatments decreased beetle survival in comparison to the corresponding acetone treatments (*p* = 0.03 and *p* = 0.02, respectively) while the third did not (*p* = 0.71, Figure 5, right column). The first Proteus 110 OD treatment resulted in a 53.8% mortality of beetles from meadows and 28.1% of beetles from the OSR fields (Table 1). The second insecticide treatment caused a 18.9% mortality of beetles from meadows and 32.8% of those from the OSR fields, and after the third treatment, the mortality was 6.7 and 11.8%, respectively (Table 1).

**Table 1:** Initial Number of Individuals and the Percentage of Mortality in Each Treatment and Habitat Type^a^

treatment/habitat type	number of individuals		mortality		comparison
	meadow	OSR	meadow	OSR	
control	40	40	15.0%	10.0%	
A-1	40	40	17.5%	12.5%	*p* = 0.46
A-2	37	57	13.5%	15.8%	*p* = 0.72
A-3	15	22	26.7%	9.1%	*p* = 0.16
P-1	160	160	53.8%	28.1%	*p* < 0.0001
P-2	37	58	18.9%	32.8%	*p* = 0.19
P-3	15	17	6.7%	11.8%	*p* = 0.58

a Symbols A-1, A-2, and A-3 represent respective acetone treatments; P-1, P-2, and P-3 represent consecutive doses of Proteus 110 OD. Control stands for nontreated individuals. Column “comparison” shows *p* values for comparisons of survival curves between habitat types within individual treatments (Wilcoxon test).

### 3.2 GUTS Analysis

**Figure 6:**
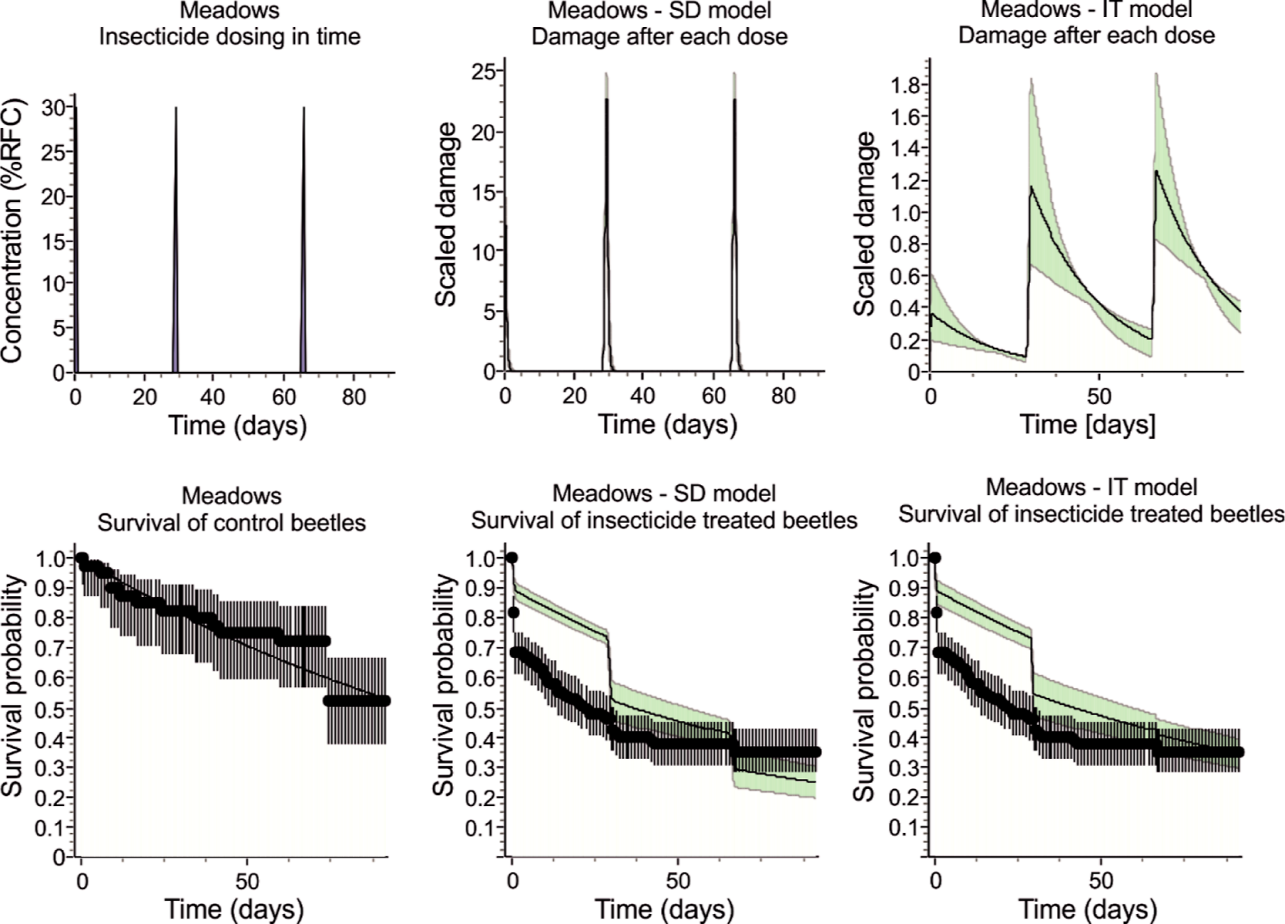
Comparison of the SD and IT openGUTS models fitted to the survival of *P. cupreus* beetles originating from meadows and exposed to three consecutive doses of Proteus 110 OD.

Both the IT and SD models failed to accurately predict the high mortality of the beetles from meadows in the first 24 h after the first pesticide dose, seriously underestimating it (Figure 6). Apparently, this resulted from undervalued damage after the first dose (Figure 6). The SD model additionally overestimated the mortality after the third dose, while the IT model predicted the final mortality accurately (Figure 6). The goodness of fit statistics do not allow us to tell clearly which model is better because generally the values of statistics did not differ substantially between the models and different statistics indicate different models: the Nash–Sutcliffe model efficiency coefficient (NSE) is 0.683 for the SD model and 0.692 for the IT model, indicating the slightly better fit of the latter. Also, the normalized root-means-square error (NRMSE; 34.4% for SD and 33.8% for IT) indicates IT model, whereas Akaike information criterion (AIC; 1200.9 for SD and 1252.5 for IT) indicates a marginally better fit of the SD model (Table 2).

**Table 2:** Comparison of the SD and IT openGUTS Models Fitted to Survival of *P. cupreus* Beetles Originating from Different Habitats, Meadows or Oilseed Rape Fields, Exposed to Three Consecutive Doses of Proteus 110 OD^b^

model parameters and goodness of fit statistics	meadows		OSR	
	SD	IT	SD	IT
*k*_d_ (95% CI)	3.24 (2.22–4.66)	0.048 (0.026–0.080)	3.19 (1.97–5.59)	0.023 (0.019–0.041)
*m*_w_ (95% CI)	5.42 × 10^–5^^a^ (5.42 × 10^–5^^a^to 1.14)	2.02 (1.14–3.47)	1.44 (5.42 × 10^–5^^a^to 4.10)	1.69 (1.16–2.94)
*b*_w_ (95% CI)	0.015 (0.011–0.020)		0.0094 (0.006–0.015)	
*F*_s_ (95% CI)		20 (6.67–20^a^)		15.97 (15.5–20^a^)
NSE	0.683	0.692	0.845	0.807
NRMSE	34.4%	33.8%	17.1%	19.2%
AIC	1200.9	1252.5	1017.2	1135.2

a Edge of 95% parameter CI reached a boundary.

b *k*_d_: dominant rate constant, *m*_w_: median of the threshold distribution, *b*_w_: killing rate, *F*_s_: spread factor of the threshold distribution, CI—confidence interval, NSE—Nash–Sutcliffe model efficiency coefficient, NRMSE—normalized root-means-square error, AIC—Akaike information criterion.

**Figure 7:**
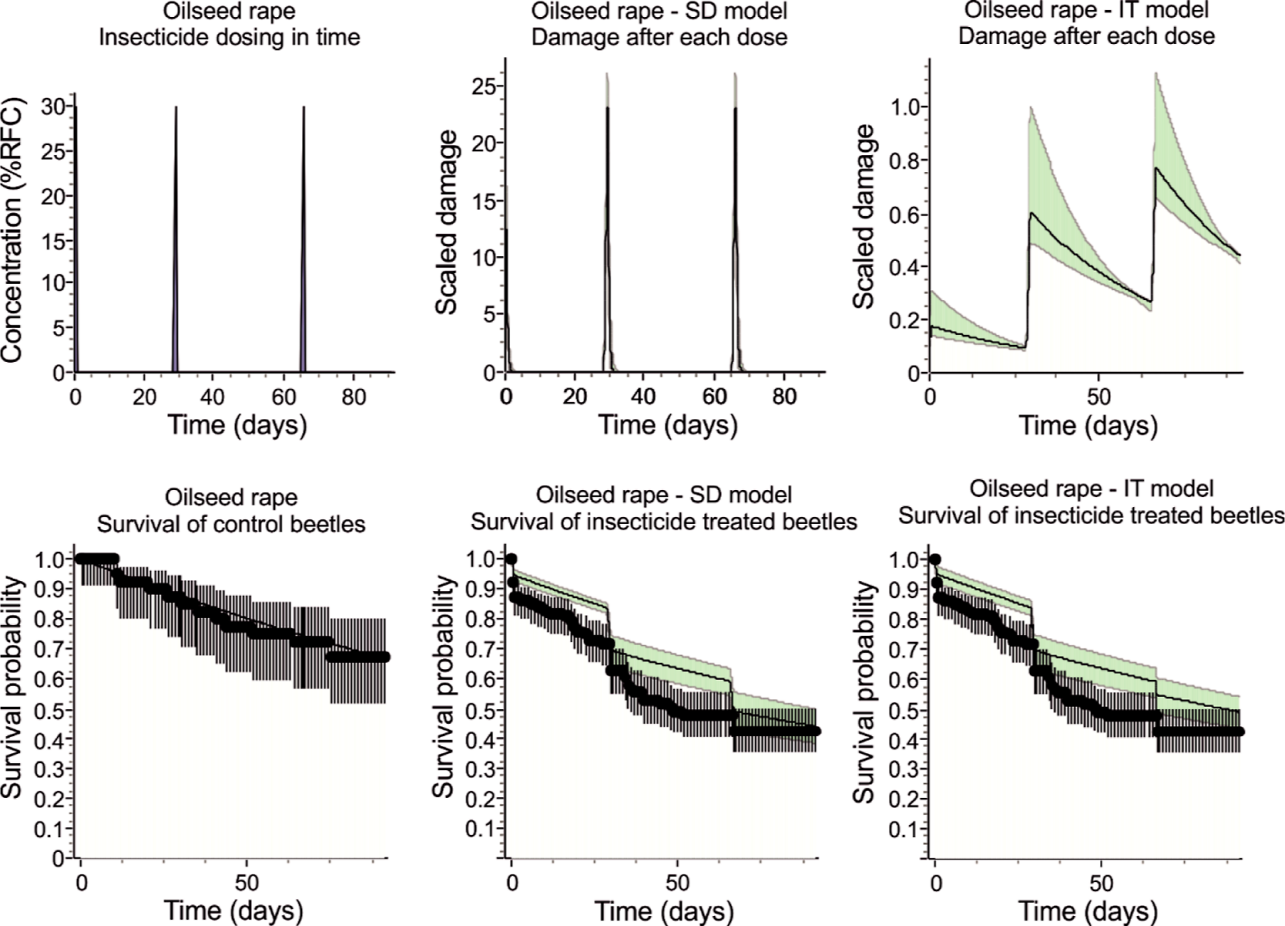
Comparison of the SD and IT openGUTS models fitted to the survival of *Poecilus cupreus* beetles originating from OSR fields and exposed to three consecutive doses of Proteus 110 OD.

For beetles from the OSR fields, the SD model is marginally better, as indicated by the NSE, NRMSE, and AIC values (Table 2). Moreover, in the case of beetles from the OSR fields, both models generally better fit the data (Figure 7), as confirmed by the higher NSE and lower NRMSE and AIC values in comparison to the respective models for beetles from meadows. This was likely due to the lack of high mortality immediately after the first treatment, which GUTS was unable to accurately model for beetles from meadows. Nevertheless, damage after the first dose was also underestimated for beetles from OSR fields, resulting in a lower modeled mortality rate than the data showed. In contrast to the beetles from the meadows, the final mortality rate of beetles from the OSR fields after three doses of the insecticide was slightly better predicted by the SD model (Figure 7).

## 4 Discussion

The results presented herein are probably the first showing that carabids may respond to repetitive exposures to insecticides differently depending on the habitat they originate from and, presumably, the history of population exposure to pesticides. The mortality rate in the beetles from meadow habitats decreased substantially with consecutive insecticide doses (from 53.8 to 18.9% to 6.7%, after the first, second, and third doses, respectively). Moreover, after the second and third treatments, the mortality patterns in insecticide-treated beetles from meadows did not differ from those in their respective solvent control groups. These results point to IT-driven mortality of beetles from meadows, as the first insecticide dose apparently selected more resistant individuals, which were then able to survive at significantly higher proportions (and not different than those in their respective solvent controls) after the next two consecutive doses of Proteus 110 OD. The overall IT-driven mortality of beetles from meadow habitats exposed to three consecutive doses of insecticide was also indicated by the slightly better fit of the GUTS-RED-IT than the GUTS-RED-SD model.

In contrast to meadow habitats, beetle mortality rates from ORC fields did not differ significantly between successive pesticide treatments and after the first dose was significantly lower than in the meadow population (28.1% vs 53.8%). This indicates that beetles collected from OSR fields could be already preselected by earlier pesticide sprays used in the sampled fields. If the earlier field spraying with pesticides killed the most sensitive individuals, the variance in sensitivity and, hence, the range of possible outcomes after the next exposures was reduced, resulting in the mortality pattern resembling stochastic mortality. The mortality of beetles from ORC fields after the second dose of insecticide was similar to that after the first dose (32.8% vs 28.1%) and was not significantly different from the meadow population after the corresponding dose (18.9%). Also, after the third dose, the mortality rate of the OSR population did not differ significantly from that of the meadow population after the same dose of the insecticide (11.8% vs 6.7%, respectively). The similar mortality rates of beetles from the OSR fields after the three consecutive insecticide treatments suggest the mortality consistent with the SD model, and their overall mortality pattern was indeed slightly better described by the GUTS-RED-SD model. Similar conclusions can be drawn thus from the Kaplan–Maier and GUTS-RED analysis: in populations with a narrow range of tolerance (such as those previously subjected to strong selection pressure in OSR habitats), the mortality pattern resembles a stochastic phenomenon. Note, however, that the stochastic mortality pattern does not mean that mortality itself is indeed a stochastic process, as this would contradict the central theory of biology—the principle of natural selection.

In the Kaplan–Meier analysis, to describe the mortality pattern correctly in both populations, no assumptions were needed about the mechanism behind mortality. Such assumptions about the nature of the death process are, however, made in the GUTS-IT and GUTS-SD models. This may lead to the erroneous conclusion that the fact that the GUTS-SD model fits the data better than the GUTS-IT model means that mortality is a stochastic process. Therefore, great care must be taken when concluding about mortality in the context of GUTS-SD assumptions. Of course, death risk depends strongly on stochastic factors, which can structure the strength and direction of selection, but these are always the organisms with more favorable traits (e.g., higher resistance to a pesticide) that have a better chance of surviving and reproducing.^45,46^

The IT model as the mechanism behind toxicants-induced mortality is consistent with such fundamental phenomena as the (log)normal distribution of individual traits in natural populations and selection through the survival of the fittest. In fact, from an evolutionary point of view, the postulation of the SD model is rather surprising, because if it were true, one would not expect rapid selection of insecticide-resistant pest populations which has been confirmed for many species (e.g., refs (47)–^48^^49^). Many observations supporting the SD model come from studies on laboratory cultures of experimental animals (e.g., refs (50)–^51^^52^), with the genetic and phenotypic variance substantially lower than in natural, wild populations. In such experiments, either the experimental animals are virtually identical (as in the case of clonal animals, such as *Daphnia magna*) and the mortality is indeed stochastic,^50^ or the variance in tolerance is too low to distinguish between the SD and IT models.^53^ Nevertheless, there are also studies in which comparable fits, or even better SD fits, were found in field-collected organisms (e.g., refs (9) and (25)). Our study seems to resolve this puzzle: the mortality pattern of the beetles from meadows was apparently driven by the varied tolerance among individuals, as indicated by particularly high mortality only after the first dose and the slightly better fit of the GUTS-IT model. In contrast, in beetles from the OSR fields, both statistical methods, i.e. Kaplan–Meier and GUTS, indicated a slightly bit better fit for the SD model. This is exactly in line with our expectation that in more genetically diverse populations from meadows, mortality should reflect differences in individual beetle tolerance, whereas in OSR populations preselected for increased pesticide tolerance, mortality may resemble a stochastic pattern.

*P. cupreus* is a spring breeder, preferring cultivated fields as its primary habitat. This leads to the majority of its population undergoing a life cycle in areas significantly impacted by ploughing and pesticide sprays. Adaptation to disturbed environments highlights the resilience of this species. In response to changing environmental conditions, populations that inhabit these cultivated fields face the need to allocate their energy resources efficiently. Consequently, these fluctuations prompt natural selection processes favoring individuals with optimized traits, resulting in the emergence of a more “robust” population. Prolonged pressure from insecticides and the scarcity of noncultivated landscape elements around cultivated fields may lead to the artificial selection of less sensitive individuals.^41^ The result would be a population with elevated average resistance to insecticides and with reduced variance in sensitivity. This could indeed produce the observed phenomenon: as the most sensitive individuals were eliminated from the population by field spraying, the mortality rate in the OSR population after the first laboratory dose was lower than that of the meadow population and resembled the stochastic pattern among the preselected individuals.

The problem with the current risk assessment, which assumes that populations are subjected to a single application event with sufficient recovery time between consecutive sprays, has been already pointed out by Brühl and Zaller.^54^ In our study, the lack of difference in survival curves after the second and third sprays between habitat types suggests that the surviving individuals had enough time to repair the damage caused by pesticide treatment. Otherwise, the carry-over effect^55^ from one spray to another would result in increasing mortality after consecutive treatments.

It should be also kept in mind that various environmental factors may act as filters sensu Hoffmann and Hercus^56^ and may be responsible for differences in the mortality rates between populations. In other words, species/populations with appropriate traits and high tolerance limits can persist,^57^ while species/populations that lack those traits^58^ or have low tolerance limits are filtered out. This may be the case here, where high mortality after the first treatment in the meadow population might be due to the presence of individuals that have not been filtered out thanks to more favorable conditions. On the other hand, the pattern of mortality in beetles from OSR fields suggests that in this case such filtration, at least partially, has already taken place. Under such nonequilibrium conditions, the environment does not necessarily impose selection on specific traits, but differences in sensitivity to a range of factors may primarily result from the underlying spatial dynamics.^59^

Generally, it may be stated that successional changes in a habitat are accompanied by modifications in the life-history patterns. Szyszko et al.,^60^ in their study on *Pterostichus oblongopunctatus*, showed that populations from different stands differed in their life history patterns. With this in mind, the data presented here suggest that habitats at more advanced stages of succession, in our case, meadows, tend to support individuals that are more diverse in their sensitivity to pesticides than less complex habitats (here, the OSR fields). In general, stressful conditions in crop fields can be extremely effective in shifting the trait averages by imposing directional selection,^61^ thereby narrowing the genetic and phenotypic variance in populations.

## Supporting Information Available

Sampling site coordinates (PDF)
Raw experimental data (XLSX)
