# Supplementary material for: Mortality Pattern of Poecilus cupreus Beetles after Repeated Topical Exposure to Insecticide—Stochastic Death or Individual Tolerance?
Source: Environ Sci Technol. 2024 Jan 22;58(4):1854–64. doi: 10.1021/acs.est.3c08031 (PMC10832044; doi:10.1021/acs.est.3c08031)
Supplement: Supplementary file 1 — es3c08031_si_001.pdf [file es3c08031_si_001.pdf]

Supporting Information for the article:

Mortality pattern of *Poecilus cupreus* beetles after repeated topical exposure to insecticide – Stochastic Death or Individual Tolerance?

Authors:

Grzegorz Sowa[1*], Agnieszka J. Bednarska[2], Ryszard Laskowski[1]

*Email: grzegorz.sylwester.sowa@gmail.com

Affiliations:

[1]Institute of Environmental Sciences, Jagiellonian University, Gronostajowa 7, 30-387 Kraków, Poland

[2]Institute of Nature Conservation, Polish Academy of Sciences, A. Mickiewicza 33, 31-120 Kraków, Poland

Pages: 2

Tables: 1

Table S1. Geographic coordinates of the sites where the beetles for the experiment were collected.

Habitat type	Coordinates (degrees)	
	Longitude	Latitude
Meadow	51.8162	17.3959
Meadow	51.7568	17.3524
Meadow	51.7249	17.2615
Oilseed rape field	51.8198	17.3252
Oilseed rape field	51.8125	17.3381
Oilseed rape field	51.7373	17.2597
